# Independent effects of acute normobaric hypoxia and hypobaric hypoxia on human physiology

**DOI:** 10.1038/s41598-022-23698-5

**Published:** 2022-11-15

**Authors:** Alejandro M. Rosales, Robert J. Shute, Walter S. Hailes, Christopher W. Collins, Brent C. Ruby, Dustin R. Slivka

**Affiliations:** 1 School of Health and Kinesiology, University of Nebraska at Omaha, Omaha, NE USA; 2 Montana Center for Work Physiology and Exercise Metabolism, Department of Integrative Physiology and Athletic Training, University of Montana, 32 Campus Drive, McGill Hall, Missoula, MT 59812 USA

**Keywords:** Physiology, Blood flow

## Abstract

The purpose of this study was to examine the effects of acute normobaric (NH, decreased FiO_2_) and hypobaric (HH, 4200 m ascent) hypoxia exposures compared to sea level (normobaric normoxia, NN). Tissue oxygenation, cardiovascular, and body fluid variables measured during rest and a 3-min step-test following 90-min exposures (NH, HH, NN). Muscle oxygenated hemoglobin (O_2_Hb) decreased, and muscle deoxygenated hemoglobin (HHb) increased environmentally independent from rest to exercise (*p* < 0.001). During exercise, brain O_2_Hb was lower at HH compared to NN (*p* = 0.007), trending similarly with NH (*p* = 0.066), but no difference between NN and NH (*p* = 0.158). During exercise, HR at NH (141 ± 4 beats·min^−1^) and HH (141 ± 3 beats·min^−1^) were higher than NN (127 ± 44 beats·min^−1^, *p* = 0.002), but not each other (*p* = 0.208). During exercise, stroke volume at HH (109.6 ± 4.1 mL·beat^−1^) was higher than NH (97.8 ± 3.3 mL·beat^−1^) and NN (99.8 ± 3.9 mL·beat^−1^, *p* ≤ 0.010) with no difference between NH and NN (*p* = 0.481). During exercise, cardiac output at NH (13.8 ± 0.6 L) and HH (15.5 ± 0.7 L) were higher than NN (12.6 ± 0.5 L, *p* ≤ 0.006) with HH also higher than NH (*p* = 0.001). During acute hypoxic stimuli, skeletal muscle maintains oxygenation whereas the brain does not. These differences may be mediated by environmentally specific cardiovascular compensation. Thus, caution is advised when equating NH and HH.

## Introduction

Decreasing the fraction of inspired oxygen (FiO_2_) at a constant barometric pressure is termed normobaric hypoxia (NH) and is frequently used to simulate altitude exposure by reducing the pressure of inspired oxygen (PiO_2_)^1^. Conversely, terrestrial altitude is termed hypobaric hypoxia (HH) and is characterized by a decrease in barometric pressure at a constant FiO_2_ to lower PiO_2_^1,2^. Using this rationale, physiological responses to a hypoxic stimulus are assumed to be dependent upon PiO_2_ regardless of whether FiO_2_ or barometric pressure are manipulated. Recent evidences challenge these assumptions, suggesting that NH and HH are not entirely equivalent^3–13^. Indeed, there is considerable debate on the physiological interchangeability of normobaric NH and HH with barometric pressure touted as a primary parameter of interest^14–17^. Exercise performance trials^5,10^, nitric oxide exhalation^6^, ventilatory responses^6,8,12^, oxidative stress markers^6^, gene expression markers^9^ and tissue oxygenation using near infrared spectroscopy (NIRS)^3^ have all been used to examine the differences between NH and HH. Disparate observations between NH and HH indeed complicate the formation of a conclusive stance on environmental interchangeability^14–17^.

The physiological responses of NH and HH exposures are well characterized; of which there are a variety commonplace findings. Across an array of exposure times (minutes-days), heart rate (HR) increases during NH and HH are common^4–6,10,11,13^ with greater increases occasionally observed during HH^12^. Similarly, blood oxygen saturation (SPO_2_) decreases during NH and HH are also common^4–6,8,13^ with larger SPO_2_ decreases occasionally observed during HH^10–12^. Additional more detailed examination of cardiac function has shown distinct differences in right heart function at rest following exercise at HH as compared to NH^13^. These works imply the presence of environmentally specific cardiovascular responses, due to atmospheric pressure decreases and not simply FiO_2_ decreases. Further work examining total body water and plasma volume changes from sea level (normobaric normoxia, NN) to terrestrial altitude (HH) commonly demonstrate decreases due to diuresis^18–21^. Although, some observations suggest that barometric pressure decreases can provoke fluid retention with HH increasing fluid retention above NH^22^. It is clear that physiological interchangeability of NH and HH warrants further examination. Observed differences among commonplace findings between NH and HH may be attributable to barometric pressure differences^14–17^.

Of recent interest has been the use of non-invasive regional tissue oxygenation assessment via NIRS^23^ to examine hypoxic environments. Indeed, exercise at NH and/or at HH produces pronounced decreases in skeletal muscle^24,25^ and brain oxygenation^24,26–28^. Muscle oxygenation has been shown to decrease during acute (minutes) and/or chronic (days) NH^24,25^ and HH^27^ exposures during exercise. Similarly, brain oxygenation also decreases during acute (minutes) and/or chronic (days) NH^24,26,28^ and HH^27^ exposures during exercise. What remains unclear from these previous works is how exactly the muscle and brain tissue behave when NH and HH are matched and compared directly. Work is needed to elucidate the independent effects FiO_2_ and barometric pressure decreases on tissue oxygenation when PiO_2_ is matched. For example, during an acute hypoxic exposure/exercise time course (~ 6–12 min), a 17% and 19% higher muscle oxygenation (tissue saturation index derived) during HH as compared to NH and NN has been shown^3^. However, logistical limitations associated with the timing of subject travel from low to high elevation precluded equivalent exposure times between NH and HH despite matched PiO_2_^3^. This is a notable limitation because physiological changes during high altitude sojourns are thought to differ as time as exposure lengthens^29^. It is thereby difficult to assert if the observed higher muscle oxygenation was attributable to FiO_2_ decrease, barometric pressure decrease, or the exposure time. Interestingly, it has also been shown that the expression of oxygen sensing genes are blunted^30^ or at least unaltered^31^ following hypoxic recovery from a single bout of exercise. Together, these findings suggest that *in-vivo* skeletal muscle may not be under hypoxic stress during acute hypoxic exposures.

Rationale for the presented work is thereby contextualized by this apparent muscle oxygenation maintenance during acute hypoxic exposures^3,30,31^. Subtle inconsistencies in regional tissue oxygenation, cardiovascular response, and body fluid observations warrant further comparison of NH and HH. The current investigation utilized a novel field-based site for its unique geographical proximity of sea level and high elevation (4200 m). This novel research site and careful attention to the study design overcame previous exposure duration limitations^3^ to better isolate FiO_2_ and barometric pressure as independent variables. Therefore, the primary purpose of this investigation was to examine how acute exposure to NH and HH during rest and exercise effect regional skeletal muscle and brain tissue oxygenation, cardiovascular variables, and body fluid.

## Methods

### Subjects

Male and female subjects who resided at approximately 975 m of elevation completed each trial (n = 18, 10 males, 8 females, 35 ± 7 years, 73.5 ± 1.7 kg, 174.9 ± 1.5 cm). Methodology was approved by the University of Montana Institutional Review Board and the Army Human Research Protections Office. Subjects were informed of the procedures and risks associated with participation prior to providing written informed consent. All procedures complied with the Declaration of Helsinki.

### Travel, testing schedule, environments, experimental overview

A field-based site was selected for its proximity and accessibility to sea level and an elevation of 4200 m. The field-based nature and short environmental exposure time required transportable data collection instruments and exercise equipment to target acute physiological responses. Subjects traveled by airplane to the field research site and were housed between trials at sea level. Subjects were tested within a consecutive 5-day period (travel to field site, 3 testing days, travel home). Repeated measures testing occurred over 3 testing days at sea level (normobaric normoxia, NN), normobaric hypoxia (NH, 12.3% FiO_2_ simulating 4200 m at sea level), and hypobaric hypoxia (HH, 4200 m) in a randomized counter-balanced order. Trials were separated by 24 h with all exposure periods lasting 90 min. Food and beverages were provided ad libitum with all subjects abstaining from alcohol during the experimental period. Prior to all testing, subjects were fasted overnight and refrained from caffeine (~ 8 h). Subjects were permitted to exercise for no greater than 60 min following testing.

During the NH trial, subjects were at sea level and inhaled incrementally lower FiO_2_ concentrations (Mag-20, Higher Peak, Stoneham, MA) adjusted every 10 min. FiO_2_ decreases were designed to mimic the gradual automobile travel required for the HH trial. During the HH trial, subjects began at NN and were driven by automobile to 4200 m in 90 min. NH and HH trials were equated in hypoxic intensity and duration, but not barometric pressure, whereas the NN and NH trials were completed at the same site, equating barometric pressure but not FiO_2_. Barometric pressure and temperature during each trial were recorded on site (Kestrel 3500 Weather Meter, Kestrel Meters, Boothwyn, PA) or supplemented from the nearest weather station. PiO_2_ was calculated using FiO_2_ (12.3%, 20.9%), barometric pressure, and an assumed water vapor pressure of 47 mmHg^16,32^. Subjects remained exposed to each environment throughout each experimental trial. All trials began with a lead in 90-min seated environmental exposure. Finger stick blood samples, a urine void, and nude body weight (Befour Inc., Cedarburg, WI) were collected immediately after the 90-min lead in period. Subjects then laid in a supine position for 15 min prior to measurements of body water. Resting cardiovascular and tissue oxygenation measurements were collected immediately following body water measurements. Subjects then completed a 3-min submaximal absolute intensity step-test exercise (39 cm, 22 steps·minute^−1^, 103.0 ± 2.3 W) with simultaneous cardiovascular and tissue oxygenation measurements in order to stimulate further demand of oxygen in the muscle. A step-test exercise was selected to target the *vastus lateralis* for its compatibility with tissue oxygenation measures (NIRS) and minimal and transportable equipment in the field.

### Tissue oxygenation

Skeletal muscle and brain oxygenation were measured using NIRS (OxyMon MKIII, Artinis, The Netherlands) as described previously^3,24–27^ for assessment of oxygenated hemoglobin (O_2_Hb) and deoxygenated hemoglobin (HHb). NIRS non-invasively measures regional tissue O_2_Hb and HHb concentrations using the relative transparency of skin at near infrared wavelengths and hemoglobin’s absorbance and scattering of near infrared light the NIRS optode distance was set at 4 cm using a black spacer adhered with double sided adhesive disks and elastic bandages. The NIRS emitter detector pairs were affixed to the distal belly of the *vastus lateralis* (15 cm superior to patella border, 5 cm lateral to thigh midline) and the forehead covering the frontal cortex region. The differential path length factor was set at 4.95 for the *vastus lateralis* and 5.93 for the brain. As all subjects resided at sea level between trials, resting baseline tissue oxygenation was measured at sea level on the day of each trial. The field-based nature and equipment constraints of this investigation required removal of the NIRS optodes between resting control and experimental conditions which could introduce more measurement variation. However, we found our resting baseline sea level average difference with these procedures to be −0.556 ± 0.565 for O_2_Hb and 0.031 ± 0.364 for HHb which were not different than “0” or no difference (*p* = 0.332 and *p* = 0.932, respectively). Resting and exercise tissue oxygenation were thereby analyzed as relative O_2_Hb and HHb concentration change from daily sea level pre-experimental environmental exposures. Tissue oxygenation was continuously measured during the above-mentioned 15-min supine rest and 3-min step-test. Tissue oxygenation analysis was completed on 15 of the 18 subjects due to equipment technical difficulties. Tissue oxygenation data were analyzed using measurements from the final 2 min of rest and steady-state exercise.

### Cardiovascular variables

SpO_2_ was measured via a finger pulse oximeter (Wrist Ox_2_ Model 3150, Nonin, Plymouth, MN). HR, stroke volume, and cardiac output were measured using non-invasive hemodynamic monitoring (Enduro, Physioflow, Poissy, France) with 6 electrodes (PF-50TM, Physioflow, Poissy France) attached at manufacturer suggested sites for resting and exercise conditions. Physioflow yields acceptable readings in healthy populations up to maximal exercise intensities^33^. Prior to each trial, all subjects were affixed with electrodes that remained adhered until data collection cessation. Electrode sites were prepped using a gel (Nuprep, Weaver and Company, Aurora, CO) and cloth by a single researcher. Indelible marker and slight residual abrasion from the gel and cloth ensured electrode placement repeatability. Blood pressure was assessed using an electronic blood pressure monitor (BP7450, Omron Healthcare Co., Kyoto, Japan). Blood pressure measurement occurred at the upper arm following the body water measurement with subjects in the supine position. SpO_2_, HR, stroke volume, and cardiac output were continuously measured during the 15-min rest and 3-min exercise. Steady state data were averaged over the final 2 min of rest and exercise for analysis.

### Body fluids

Finger-stick blood samples (10 µL) were collected for immediate hemoglobin measurement (Hb 201 + , HemoCue, Brea, CA). An additional finger-stick blood sample was collected into a capillary tube (60–100 µL) for hematocrit measurement. Capillary tube samples were immediately centrifuged at 12,000 RPM (Zip Combo, LW Scientific, Lawrenceville, GA) for 3 min. Hemoglobin and hematocrit measurements were used for quantification of plasma volume alterations^34^. Similar to the NIRS measurements, plasma volume was normalized to the pre-exposure NN trial for comparison between environments. A urine sample was used to measure urine specific gravity (USG) via a calibrated refractometer (PAL-10S, Atago, Tokyo, Japan). USG was analyzed in 17 subjects as 1 subject was unable to provide a urine sample. Total body water, extracellular fluid, and intracellular fluid were measured via bioelectrical impedance (s10, InBody USA, Cerritos, CA) after 15 min of supine rest per manufacturer recommendations.

### Statistical analysis

Barometric pressure, ambient temperature, PiO_2_, body fluids, and blood pressure were analyzed using a one-way repeated measures analysis of variance (environment). Mauchly’s test of sphericity was not violated during any one-way repeated measures analysis of variance. Cardiovascular variables and tissue oxygenation were analyzed using a two-way repeated measure analysis of variance (environment x exercise). Statistical significance was set at a type I probability error of < 5% (*p* < 0.05), with data expressed as means ± SE. Statistical analysis was completed using SPSS (IBM, Chicago, IL).

## Results

### Environmental conditions

Barometric pressure at HH (464.8 ± 3.8 mmHg) was significantly lower than NH (757.2 ± 0.5 mmHg) and NN (754.4 ± 3.0 mmHg) (*p* < 0.001) but did not significantly differ between NH and NN (*p* = 0.379). Ambient temperature at HH (18.3 ± 1.2 °C) was significantly lower than NH (27.7 ± 0.2 °C) and NN (27.7 ± 0.1 °C) (*p* < 0.001) but did not significantly differ between NH and NN (*p* = 0.553). PiO_2_ showed no significant differences between HH (87.3 ± 0.8 mmHg) and NH (88.1 ± 0.1 mmHg) (*p* = 0.360). Accordingly, the PiO_2_ at HH and NH were significantly lower than NN (147.8 ± 0.6 mmHg) (*p* < 0.001).

### Tissue oxygenation

**Figure 1:**
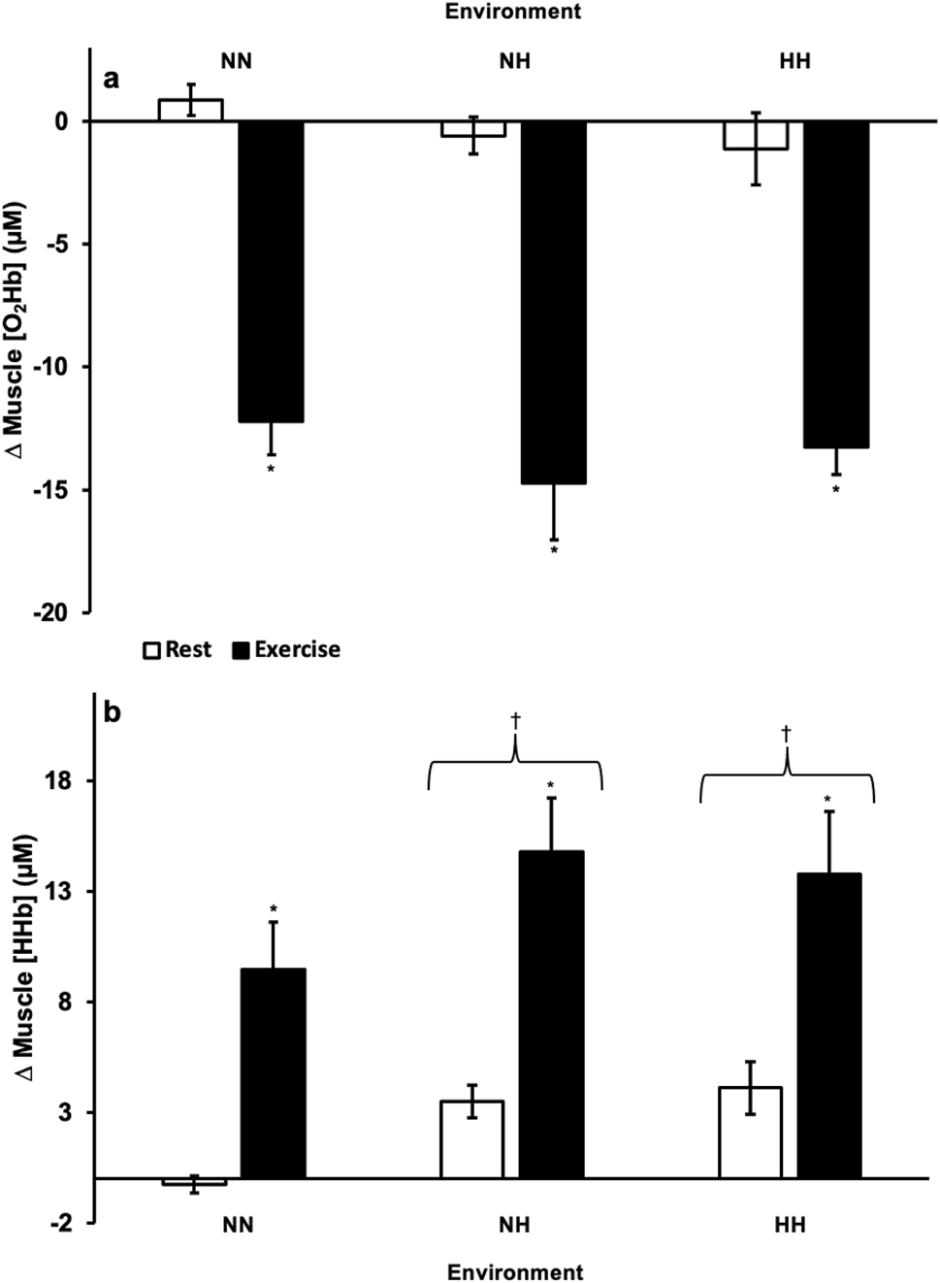
(**a**) Muscle oxygenated (O_2_Hb) and (**b**) deoxygenated (HHb) hemoglobin concentration change at normobaric normoxia (NN), normobaric hypoxia (NH), and hypobaric hypoxia (HH) during rest and exercise expressed relative to daily pre-environmental exposure at NN. *p < 0.05 from Rest. †*p* < 0.05 from NN. Data presented as means ± SE.

Muscle O_2_Hb was not significantly different between HH, NH, and NN at rest or during exercise (*p* = 0.339) (Fig. 1a). However, within each environment, muscle O_2_Hb was significantly lower during exercise than at rest (*p* < 0.001) (Fig. 1a). Conversely, muscle HHb was significantly higher during the HH and NH exposures compared to NN (*p* = 0.001, *p* < 0.001, respectively) but not significantly different between the HH and NH exposures (*p* = 0.861) (Fig. 1b). Within each environment, muscle HHb was significantly higher during exercise than at rest (*p* < 0.001) (Fig. 1b).

**Figure 2:**
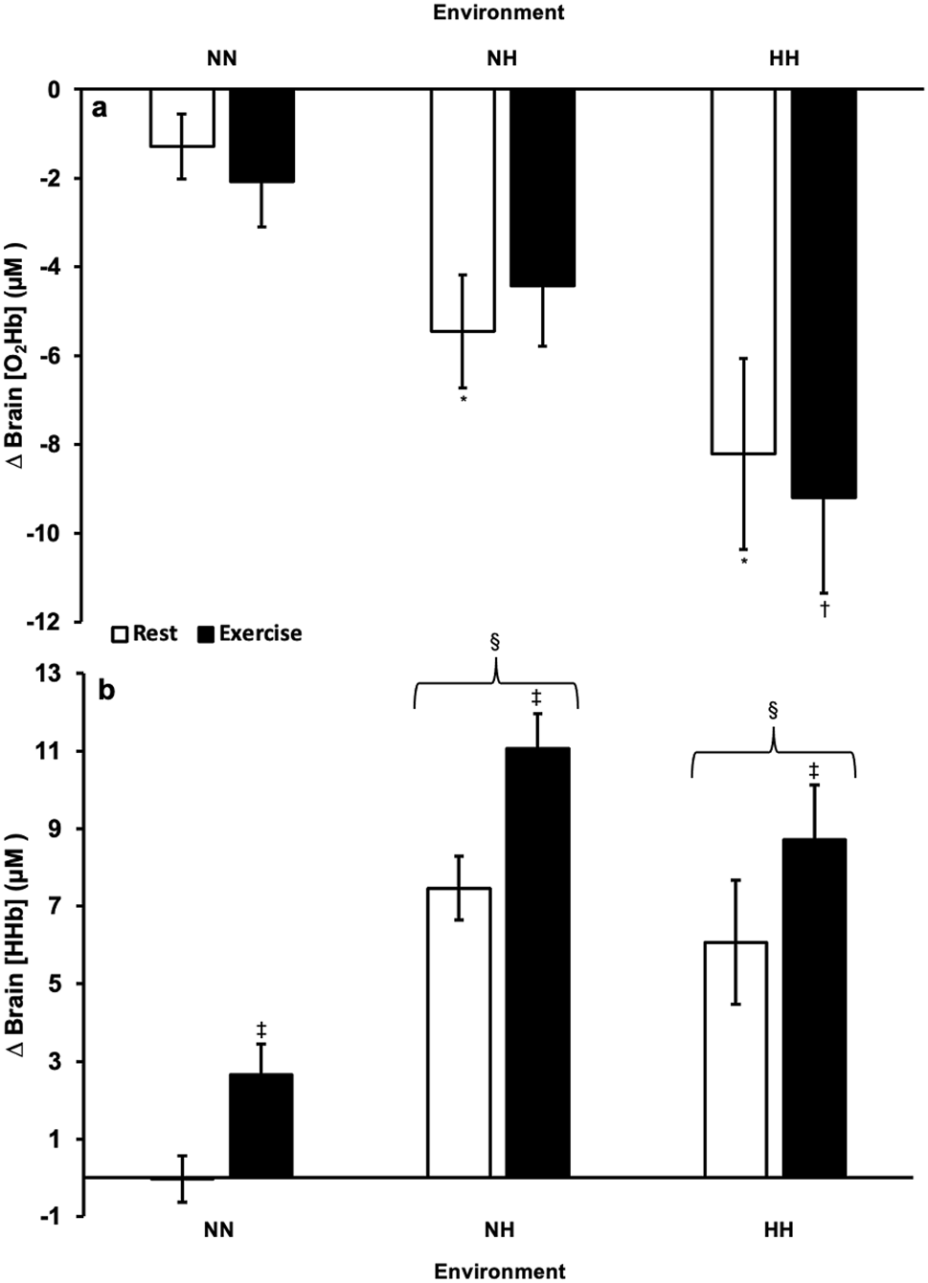
(**a**) Brain oxygenated (O_2_HB) and (**b**) deoxygenated (HHb) hemoglobin concentration change at normobaric normoxia (NN), normobaric hypoxia (NH), and hypobaric hypoxia (HH) during rest and exercise expressed relative to daily pre-environmental exposure at NN.*p < 0.05 from Rest at NN. †*p* < 0.05 from Exercise at NN. ‡*p* < 0.05 from Rest. §*p* < 0.05 from NN. Data presented as means ± SE.

Resting brain O_2_Hb was significantly lower during the HH and NH exposures than NN (*p* = 0.009, *p* = 0.012, respectively), but not significantly different between the HH and NH exposures (*p* = 0.278) (Fig. 2a). Moreover, brain O_2_Hb during exercise was significantly lower during the HH exposure than NN (*p* = 0.007) but was not significantly different between the NH exposure and NN (*p* = 0.158) (Fig. 2a). There was a similar trend noted for brain O_2_Hb to be significantly lower during exercise at HH compared to NH (*p* = 0.066) (Fig. 2a). Conversely, brain HHb was significantly higher during the HH and NH exposures compared to NN (*p* = 0.001, *p* < 0.001), but not significantly different between HH and NH exposures (*p* = 0.223) (Fig. 2b). Within each environment, brain HHb during exercise was significantly higher than at rest (*p* < 0.001) (Fig. 2b).

### Cardiovascular variables

Systolic blood pressure at rest was significantly higher during the HH exposure than NN (*p* < 0.001) (Table 1). There were similar trends noted for resting systolic blood pressure to be significantly higher during the NH exposure compared to NN (*p* = 0.055) as well as a significantly higher systolic blood pressure during the HH exposure compared to the NH exposure (*p* = 0.053) (Table 1). Diastolic blood pressure at rest was significantly higher during the HH exposure than NN (*p* = 0.020), but not different than NH (*p* = 0.335) (Table 1). Although, diastolic blood pressure at rest during the NH exposure was not significantly different than NN (*p* = 0.106) (Table 1). SpO_2_ was significantly lower during the HH and NH exposures than NN (*p* < 0.001) but was not significantly different between the HH and NH exposures (*p* = 0.208) (Table 1). Although, SpO_2_ during exercise was significantly higher than at rest within each environment (*p* = 0.010) (Table 1).

**Table 1:** Cardiovascular variables at rest and exercise following acute exposure to normobaric normoxia (NN), normobaric hypoxia (NH), and hypobaric hypoxia (HH). *p < 0.05 from Rest. †*p* < 0.05 from NN. ‡*p* < 0.05 from NH. Data presented as means ± SE.

	NN		NH		HH	
	Rest	Exercise	Rest	Exercise	Rest	Exercise
Systolic (mmHg)	110 ± 2	–	114 ± 2	–	117 ± 3†	–
Diastolic (mmHg)	64 ± 1	–	66 ± 1	–	67 ± 1†	–
SPO_2_ (%)	96 ± 0.4	97 ± 0.4*	79 ± 1.4†	82 ± 1.0*†	79 ± 1.4†	80 ± 0.9*†
Heart rate (beats·minute^-1^)	61 ± 2	127 ± 44*	70 ± 2†	141 ± 4*†	72 ± 3†	141 ± 3*†
Stroke volume (mL·beat^-1^)	102.2 ± 3.1	99.8 ± 3.9	95.0 ± 4.1†	97.8 ± 3.3	96.5 ± 3.7	109.6 ± 4.1*†‡
Cardiac output (L·minute^-1^)	6.2 ± 0.2	12.6 ± 0.5*	6.6 ± 0.3	13.8 ± 0.6*†	6.8 ± 0.3†	15.5 ± 0.7*†‡

Resting HR was significantly higher during the HH and NH exposures than NN (*p* < 0.001, *p* = 0.002, respectively) while resting HR was not significantly different between the HH and NH exposures (*p* = 0.397) (Table 1). Furthermore, HR during exercise was significantly higher than at rest within each environment (*p* < 0.001) (Table 1). Accordingly, HR during exercise was significantly higher during the HH and NH exposures than NN (*p* < 0.001, *p* < 0.001, respectively) whereas HR during exercise at HH was not significantly different than NH (*p* = 0.790) (Table 1). Resting stroke volume was significantly lower during the NH exposure than NN (*p* = 0.044), with only a trend for a significantly lower resting stroke volume between the HH exposure and NN noted (*p* = 0.073) (Table 1). Resting stroke volume was not significantly different between the HH and NH exposures (*p* = 0.704) (Table 1). Stroke volume during exercise was not significantly different from rest at NN (*p* = 0.465) or during the NH exposure (*p* = 0.502) (Table 1). However, stroke volume during exercise was significantly higher than rest during the HH exposure (*p* = 0.005) (Table 1). Moreover, stroke volume during exercise was significantly higher during the HH exposure than NN (*p* = 0.010) and the NH exposure (*p* = 0.002) (Table 1). Although, stroke volume during exercise was not significantly different between the NH exposure and NN (*p* = 0.481) (Table 1). Resting cardiac output was significantly higher during the HH exposure than NN (*p* = 0.004), but not different than NH (*p* = 0.224) (Table 1). Resting cardiac output during the NH exposure was not significantly different than NN (*p* = 0.125) (Table 1). Cardiac output during exercise was significantly higher than at rest within each environment (*p* < 0.001) (Table 1). Moreover, cardiac output during exercise was significantly higher during the HH and NH exposures than NN (*p* < 0.001, *p* = 0.006, respectively) (Table 1). Additionally, cardiac output during exercise was significantly higher during the HH exposure than the NH exposure (*p* = 0.001) (Table 1).

### Nude body weight and body fluids

Nude body weight did not significantly differ across environments (*p* = 0.536) (Table 2). Hemoglobin was significantly higher during the HH exposure compared to NN (*p* < 0.001) and the NH exposure (*p* = 0.005) with a trend for hemoglobin to be significantly lower during the NH exposure than NN (*p* = 0.052) (Table 2). Similarly, hematocrit was significantly higher during the HH exposure compared to NN (*p* < 0.001) and the NH exposure (*p* < 0.001) (Table 2). There was no difference in hematocrit between the NH exposure and NN (*p* = 0.255) (Table 2). Plasma volume normalized relative to NN was significantly lower during the HH exposure compared to the NH exposure (*p* < 0.001) (Table 2). USG was not significantly different between environments (*p* = 0.169) (Table 2). Total body water, extracellular fluid, and intracellular fluid were significantly lower during the HH exposure compared to NN (*p* < 0.001, *p* < 0.001, and *p* = 0.001, respectively), not significantly different between the NH exposure and NN (*p* = 0.492, *p* = 0.696, and *p* = 0.420, respectively), and significantly lower during the HH exposure compared to the NH exposure (*p* = 0.006, *p* = 0.006, and *p* = 0.016, respectively) (Table 2).

**Table 2:** Body weight, hemoglobin, hematocrit, plasma volume, urine specific gravity, and body water following acute exposure to normobaric normoxia (NN), normobaric hypoxia (NH), and hypobaric hypoxia (HH). *p < 0.05 from NH and NN. Data presented as means ± SE.

	NN	NH	HH
Body weight (kg)	73.5 ± 3.0	73.4 ± 2.9	73.5 ± 2.9
Plasma volume (% change from sea level)	–	− 3.8 ± 2.2	− 16.6 ± 2.0*
Hemoglobin (g·dL^-1^)	14.2 ± 0.3	14.7 ± 0.3	15.5 ± 0.4*
Hematocrit (%)	42.5 ± 0.9	43.2 ± 0.9	48.3 ± 0.8*
Urine specific gravity	1.014 ± 0.002	1.015 ± 0.002	1.012 ± 0.002
Total body water (L)	43.8 ± 1.8	43.7 ± 1.8	43.0 ± 1.8*
Extracellular fluid (L)	16.4 ± 0.7	16.3 ± 0.7	16.1 ± 0.7*
Intracellular fluid (L)	27.4 ± 1.1	27.3 ± 1.2	27.0 ± 1.1*

## Discussion

The present investigation utilized the natural geography of a field-based research site to quickly (< 90 min) expose subjects to HH in comparison to NH and NN. This investigation matched the duration and PiO_2_ of hypoxic stimuli during NH and HH to examine independent effects of barometric pressure and FiO_2_ on regional skeletal muscle and brain tissue oxygenation, cardiovascular variables, and body fluid. The primary findings of this study were that during acute NH, HH, and NN, skeletal muscle O_2_Hb was not different between environments, but brain O_2_Hb was lower during the NH and HH exposures than the NN condition. Thus, indicating similar oxygen availability to the muscle, but not the brain, during NH and HH as compared to NN. HHb was higher during NH and HH in both muscle and brain. The higher amount of HHb without an alteration in O_2_Hb may indicate sufficient O_2_ availability via enhanced blood flow to these tissues. Indeed, cardiac output during exercise was higher in the NH and HH conditions compared to the NN condition, which provides a mechanism for enhanced blood delivery, albeit via indirect methods. Interestingly, the mechanism by which the magnitude of cardiac output is increased appears environmentally dependent. Specifically, during exercise, HR appears to be the main contributor to increased cardiac output during NH, whereas further cardiac output increases at HH rely upon enhanced stroke volume. These cardiac alterations seemingly compensate for decreased SPO_2_ and allow skeletal muscle to be relatively normoxic. However, these cardiovascular compensations do not appear to preserve the oxygenation of the brain. These findings propose that acute NH and HH are not interchangeable environments. Decreases in FiO_2_ may not satisfactorily simulate all physiological outcomes experienced from barometric pressure decreases at terrestrial altitude.

Overcompensated skeletal muscle oxygenation during graded exercise to volitional fatigue at HH when compared to NH and NN has been observed^3^. However, logistical limitations confounded hypoxic exposure time between NH and HH. Although, blunted^30^ or at least unaltered^31^ expression of the oxygen sensing gene hypoxia inducible factor 1α following exercise (70% NN max cycling power) and hypoxic recovery (4–6 h) has also been observed. Together these works suggest adequate skeletal muscle oxygenation under acute hypoxic exposures. During exercise, muscle oxygenation measured at the vastus lateralis is reported to decrease under both hypoxic and normoxic conditions^3,24,25,27^. Muscle oxygenation decreases have been found during incremental/ramped type exercise protocols^3,24,27^ and sprints^25^ during hypoxia (3000–4300 m or equivalent FiO_2_) are of different duration and intensity than the step-test exercise used here. Muscle oxygenation decreases may therefore be a part of the normal exercise response as inspiration of oxygen during maximal cycling exercise at 4300 m or an equivalent FiO_2_ does not meaningfully increase muscle oxygenation compared to NH^27^. Oxygen demand at the muscle may be maintained during acute hypoxic exposures. However, the magnitude of muscle oxygenation decrease between hypoxic and normoxic environments is at times variable. Hypoxic environments have been shown to elicit lower^3,24,25^ and similar^3,27^ muscle oxygenation decreases as compared to normoxic environments. In the current study, muscle oxygenation decreases due to exercise were not different between acute NH, HH, and NN. These similar muscle oxygenation responses at the skeletal muscle level support a common exercise response as opposed to an environmentally specific response. These findings lend credence to maintained or at least unaltered skeletal muscle gene expression responses to hypoxia^30,31^.

Our observed brain tissue alterations did not respond in a similar fashion to muscle oxygenation. Brain oxygenation decreased under both tested hypoxic derivations. Resting brain oxygenation at NH and HH were not different but were lower than NN. Previous works have shown similar findings at 11–14% FiO_2_^26,28^. Brain tissue oxygenation and/or blood flow changes are often targeted as a critical factor in evaluating/regulating exercise fatigue or hypoxic brain protection^26–28^. During incremental exercise with simultaneous NIRS measurements at the frontal cortex, identical sites to the present study, acute (1-h) and chronic (5-day acclimation) HH exposures of 4300 m decreased brain oxygenation compared to NN^27^. Interestingly, brain oxygenation decreases at HH can be instantaneously reversed upon inspiration of concentrated oxygen at maximal exercise^27^. Peripheral chemoreception and subsequent oxygen delivery to the brain therefore appears sensitive to hypoxic stimuli regardless of the duration and exposure type. The permitted decreases in brain tissue oxygen thereby suggest functional tolerance against a spectra of oxygenation levels from a resultant PiO_2_ decrease^24^, which supports our brain oxygenation decreases at NH and HH. Importantly, we did observe marked brain O_2_Hb decreases during exercise at HH when compared to NN as well as a similar trend for brain O_2_Hb decrease at HH compared to NH, which may point to a barometric pressure effect. Cerebral oxygen delivery may actually decrease at lower barometric pressures due to blunted cerebrovascular reactivity^35^.

In accordance with our findings, simultaneous examination of muscle and brain oxygenation at rest and short steady-state cycling at NH (FiO_2_ 12–14%) muscle oxygenation is maintained, and brain oxygenation is decreased as compared to NN^26^. With decreased PiO_2_ under hypoxic conditions, it seems anticipated that the muscle may require increased oxygen delivery. Increased sympathetic tone and vagal withdrawal has been demonstrated at rest when exposed to HH^4^ prior to the sympathetic effect of exercise. Exercising muscle will demand appropriately matched oxygen delivery to accommodate the metabolic demand of working tissue^36^. Conversely, cerebral metabolism does not significantly change during rest or exercise at 100 W following acclimatization to 5200 m^37^ which can be accompanied by barometrically induced cerebral oxygen delivery decreases^35^. The subjects in the presented work exercised at ~ 100 W and our muscle and brain oxygenation responses may have arisen from the anticipated need to increase oxygen delivery to working tissue over the 90-min exposure coupled with maintained brain metabolism despite decreased oxygen delivery.

Muscle and brain tissue oxygenation responses may be driven by environmentally distinct cardiovascular responses. It is commonly accepted that relative exercise intensity at a given workload increases under hypoxic conditions when compared to NN due to decreases in maximal exercise capacity^38^. The current study utilized an absolute intensity step-test exercise across each environment with greater relative exercise intensities likely experienced at NH and HH as compared to NN. Despite this difference, is it unlikely that relative exercise intensity differed between NH and HH since our investigation showed similar PiO_2_, HR, and SpO_2_ responses between NH and HH during rest and exercise. Together this confirms similar magnitude of hypoxic stimuli. Similar HR and SpO_2_ responses are not an uncommon observation between matched NH and HH^3,5,6,8^. SpO_2_ decreases are associated with HR mediated cardiac output increases during exercise^5^, but this only partially contributes to our observed HH induced cardiac output increase during exercise as compared to NH.

During exercise at HH when compared to NH, despite similar HR increases, there were additional stroke volume increases. Historically, stroke volume increases are accepted up to 40% maximal exercise capacity^39^, however recent analyses do suggest increases up to 100% maximal exercise capacity are attainable^40^. Stroke volume increases could have been mediated by blood pressure alterations. We observed an HH induced increase in resting systolic and diastolic blood pressure compared to NN. Systolic blood pressure at HH also trended higher than NH. Blood pressure measurements following acute terrestrial altitude ascents (≤ 24 h, 3700–4900 m) have shown increased systolic and diastolic blood pressures compared to sea level at rest^41,42^, with exacerbated systolic blood pressure following a step-test^41^ similar to the one used here. Moreover, carotid baroreceptor function at HH is preserved but may not be potent enough to counteract blood pressure increases at altitude^36^. Increased systolic blood pressure carried over from the rest to exercise transition at HH may reflect an increased end diastolic volume and concomitant stroke volume increase via the Frank-Starling mechanism^43^. However, cardiac output increases without stroke volume increases have been observed 15 min following 2 h of exercise at HH^13^ with some suggesting that the capacity to increase stroke volume during HH is impaired compared to NN^44^. Regardless, the presently observed environmentally driven cardiovascular compensations during exercise at HH may be potent enough to maintain the above-mentioned relatively normoxic skeletal muscle oxygenation, but not brain oxygenation.

Total body water, extracellular fluid, intracellular fluid, and plasma volume decreases are commonly reported after terrestrial altitude ascent from sea level after ≥ 2 days due to increased diuresis^18–21^. Body fluid decreases are most often ascribed to an increased respiratory water loss and/or urinary output^19,20,29^. It is thereby expected that our observed body water losses at HH reflect both respiratory water loss and urine output avenues. A focal point of this investigation was the matched acute exposure duration with independent isolation of FiO_2_ and barometric pressure variables. The difference between HH and NH was barometric pressure and the difference between NN and NH was FiO_2_. If body water changes were simply a response to decreased PiO_2_, then it would be expected that our NH and HH environments would not have differed. This investigation found a decrease in body fluid at HH when compared to NN and NH, whereas NN and NH did not showcase body water differences. These differences were further evinced in plasma volume decreases driven by fluid loss/hemoconcentration as derived from hemoglobin and hematocrit measurements^34^. However, increased fluid retention has been observed following 10 h of HH compared to an equivalent NH with this response attributable to increased acute mountain sickness risk^22^. In any event, alterations in barometric pressure^45–47^ and local pressure^42^ appear transmittable across a variety of human biological tissue. Oncotic pressure differences have also been estimated during HH, with increases occurring as early as 24 h into a terrestrial altitude sojourn (4200 m)^48^. The acute barometric pressure decrease experienced at HH may provide the early stimulus for commonly observed acclimatization dependent body water changes^18,20^.

## Conclusions

Decreases in FiO_2_ are often used to simulate decreases in PiO_2_ experienced during high elevation sojourns^1^. This investigation isolated barometric pressure and FiO_2_, clamping duration and intensity of hypoxic stimuli between NH and HH. Our findings propose that during acute hypoxic stimuli, skeletal muscle is not differentially hypoxic across environments whereas brain oxygenation is. These differences are mediated by environmentally driven cardiovascular compensation. Specifically, during exercise, HR is the main contributor to increased cardiac output during NH, with further cardiac output increases at HH relying upon enhanced stroke volume. Caution is warranted when interchanging acute NH and HH. The physiological changes associated with barometric pressure differences are not entirely matched through FiO_2_ manipulation. However, our unique study design only provides insight into the very acute phase of hypoxic exposure which may adjust in an acclimation dependent manner.

## Data Availability

Data presented in this manuscript has not been deposited into public repositories but may be available upon request from the corresponding author.

## Abbreviations

HH: Hypobaric hypoxia
FiO_2_: Fraction of inspired oxygen
HHb: Deoxygenated hemoglobin
HR: Heart rate
NH: Normobaric hypoxia
NIRS: Near infrared spectroscopy
O_2_Hb: Oxygenated hemoglobin
PiO_2_: Pressure of inspired oxygen
NN: Sea level
SpO_2_: Blood oxygen saturation
USG: Urine specific gravity
